# Factors Affecting Temporal Changes in Ablated Liver Volume After Radiofrequency Ablation for Hepatocellular Carcinoma Evaluated by Three-Dimensional Volumetric Computed Tomography

**DOI:** 10.3390/tomography12080112

**Published:** 2026-08-07

**Authors:** Mayumi Higashi, Masahiro Tanabe, Yuna Fujii, Haruki Furutani, Jo Ishii, Yuto Takemura, Norikazu Tanabe, Issei Saeki, Taro Takami, Katsuyoshi Ito

**Affiliations:** 1 Department of Radiology, Yamaguchi University Graduate School of Medicine, 1-1-1 Minami-Kogushi, Ube 755-8505, Yamaguchi, Japanj-ishii@yamaguchi-u.ac.jp (J.I.);; 2 Department of Gastroenterology and Hepatology, Yamaguchi University Graduate School of Medicine, 1-1-1 Minami-Kogushi, Ube 755-8505, Yamaguchi, Japan; norikazu@yamaguchi-u.ac.jp (N.T.); t-takami@yamaguchi-u.ac.jp (T.T.)

**Keywords:** computed tomography, liver, radiofrequency ablation, liver function, liver fibrosis

## Abstract

Objectives: To evaluate associations between shrinkage of ablated liver area on computed tomography (CT) over time after radiofrequency ablation (RFA) and clinical parameters related to liver function and fibrosis. Methods: Patients with hepatocellular carcinoma who underwent RFA and follow-up CT were retrospectively reviewed. The ablated area volume (AAV) on CT obtained within 1 week and around 6 months after RFA was measured, and reduction rate of AAV was calculated. The AAV reduction rate was compared among Child-Pugh classification, modified albumin-bilirubin (mALBI) grades, FIB-4 index categories, and lesion locations using generalized estimating equations (GEE). Univariable and multivariable GEE analyses were performed to evaluate associations between the AAV reduction rate and clinical parameters. Results: Fifty-three lesions in 41 patients (median age, 76 [range, 38–88] years, 24 men) were evaluated. The AAV reduction rate was significantly lower in Child-Pugh class B than class A (*p* < 0.001) and in mALBI grade 2b than grade 1 (*p* < 0.001) or grade 2a (*p* = 0.004). Significant differences were also observed among FIB-4 index groups (*p* < 0.001) and among lesion locations, with lower AAV reduction rates in medial and anterior segments than in lateral (*p* < 0.001) and posterior (*p* = 0.017) segments. Univariable GEE analyses showed significant associations between AAV reduction rate and cholinesterase, albumin, total bilirubin (T-Bil), prothrombin time, platelet count, and FIB-4 index (*p* < 0.05). Multivariable GEE analysis demonstrated that both albumin and T-Bil remained independently associated with AAV reduction rate (*p* < 0.001). Conclusions: The AAV reduction rate tended to be lower in patients with impaired liver function and advanced liver fibrosis.

## Simple Summary

Radiofrequency ablation is a common treatment for hepatocellular carcinoma, and the ablation zone gradually shrinks on follow-up computed tomography. However, the degree of shrinkage varies considerably among patients. This study evaluated whether this variability was associated with liver function and liver fibrosis using three-dimensional volumetric analysis. Limited shrinkage of the ablation zone was associated with impaired liver function and advanced liver fibrosis. These findings may help radiologists better interpret follow-up computed tomography after radiofrequency ablation.

## 1. Introduction

Radiofrequency ablation (RFA) of the liver has been established as a standard treatment for early stage hepatocellular carcinoma (HCC) and small liver metastases, in patients who are unsuitable candidates for surgical resection because of limited hepatic functional reserve, significant comorbidities, or a poor general condition [1,2,3,4,5]. The procedure is typically performed percutaneously under image guidance by inserting the electrode directly into the tumor and applying radiofrequency energy to induce coagulative necrosis of the targeted tissue [6]. Owing to its safety, efficacy, reproducibility, and low complication rate, RFA has been increasingly adopted in clinical practice [7].

Contrast-enhanced computed tomography (CT) is the most widely used modality for follow-up evaluation of the treatment response after RFA [8]. On follow-up CT, the ablation zone in the liver typically appears as a non-enhancing low-attenuation area, representing coagulative necrosis, and its size generally decreases gradually over time. A previous study demonstrated that the ablated liver volume decreased by approximately 50% of that on the immediate follow-up CT scans at 4 months after RFA and by more than 90% at 19 months after RFA [9]. However, the degree of shrinkage of the ablated liver area varies considerably between patients. In some cases, the ablation zone diminishes markedly, whereas in others, it shows only a minimal reduction and persists for a prolonged period. The clinical factors responsible for this variability have not yet been fully elucidated.

Therefore, the purpose of this study was to evaluate the association between the degree of reduction in the ablated liver volume on follow-up CT after RFA and clinical parameters including laboratory data related to the liver function and liver fibrosis.

## 2. Materials and Methods

### 2.1. Study Population

This retrospective study was approved by our institutional review board, which waived the need for written informed consent. A search of our hospital’s radiology reporting database system was performed to identify patients suspected of having HCC based on ultrasonography, CT, or magnetic resonance imaging (MRI) findings, and who underwent RFA and follow-up abdominal CT examinations at our hospital between June 2015 and October 2023. The inclusion criteria were as follows: (a) patients who underwent contrast-enhanced multiphasic dynamic CT and (b) follow-up CT examinations performed within 1 week and around 6 months after RFA. Patients who underwent concurrent transcatheter arterial chemoembolization were excluded from the study.

### 2.2. Clinical Data

Clinical and laboratory data at the time of each RFA procedure were recorded. In patients who underwent RFA for multiple lesions at different time points, laboratory values corresponding to each RFA session were used for lesion-based analyses. The clinical information included age, sex, a history of prior HCC treatments, ascites, and hepatic encephalopathy. Laboratory data included aspartate aminotransferase (AST), alanine aminotransferase (ALT), lactate dehydrogenase (LDH), alkaline phosphatase (ALP), cholinesterase (ChE), albumin, total bilirubin (T-Bil), direct bilirubin (D-Bil), prothrombin time, and platelet counts. The interval between the first and second follow-up CT examinations was also recorded. The fibrosis-4 (FIB-4) index, an indicator of liver fibrosis, was calculated using the following formula: (age [years] × AST [IU/L])/(platelet count [10^9^/L] × (ALT [IU/L])^1/2^) [10]. The patients were categorized into three groups based on the low (1.3) and high (2.67) cutoff values of the FIB-4 index for the assessment of fibrosis risk [11] as follows: FIB-4 < 1.3 (low), FIB-4 1.3 to ≤2.67 (indeterminate), and FIB-4 > 2.67 (high). In addition, the Child-Pugh classification and modified albumin-bilirubin (mALBI) grade were assessed based on the clinical data to estimate the hepatic functional reserve [12,13]. According to the Child-Pugh score, calculated using serum concentrations of T-Bil and albumin, prothrombin time, and the degree of ascites and hepatic encephalopathy [12], the patients were divided into two categories: Child-Pugh A and Child-Pugh B. The ALBI score was calculated using the following formula: (log_10_ T-Bil [µmol/L] × 0.66) + (albumin [g/L] × −0.085) [14], and the mALBI grade was defined by the ALBI score as follows: grade 1, ≤−2.60; grade 2a, >−2.60 to <−2.27; grade 2b, −2.27 to ≤−1.39; and grade 3, >−1.39 [13].

### 2.3. CT Technique

All CT imaging scans were performed using multi-detector CT scanners from multiple vendors (Somatom Definition, Somatom Force, or Somatom Sensation, Siemens, Erlangen, Germany; Optima CT660, GE Healthcare, Milwaukee, WI, USA; Aquilion Precision, Canon Medical Systems, Otawara, Japan). The imaging parameters were as follows: tube voltage, 100–110 kVp for the Somatom Force and 120 kVp for the others with modulated tube current; matrix size, 512 × 512; and reconstruction interval, 1.0–1.25 mm. The multiphasic contrast-enhanced CT protocol was as follows: after the acquisition of unenhanced images, triple-phase contrast-enhanced dynamic CT was performed following a bolus injection of approximately 600 mg I/kg of nonionic contrast material with an injection time of 30 s (the injection rate was 3–5 mL/s, depending on the patient’s weight). Arterial-phase, portal-phase, and equilibrium-phase images were obtained with delays of 40, 70, and 180 s after injection of the contrast material, respectively.

### 2.4. Image Analyses

**Figure 1:**
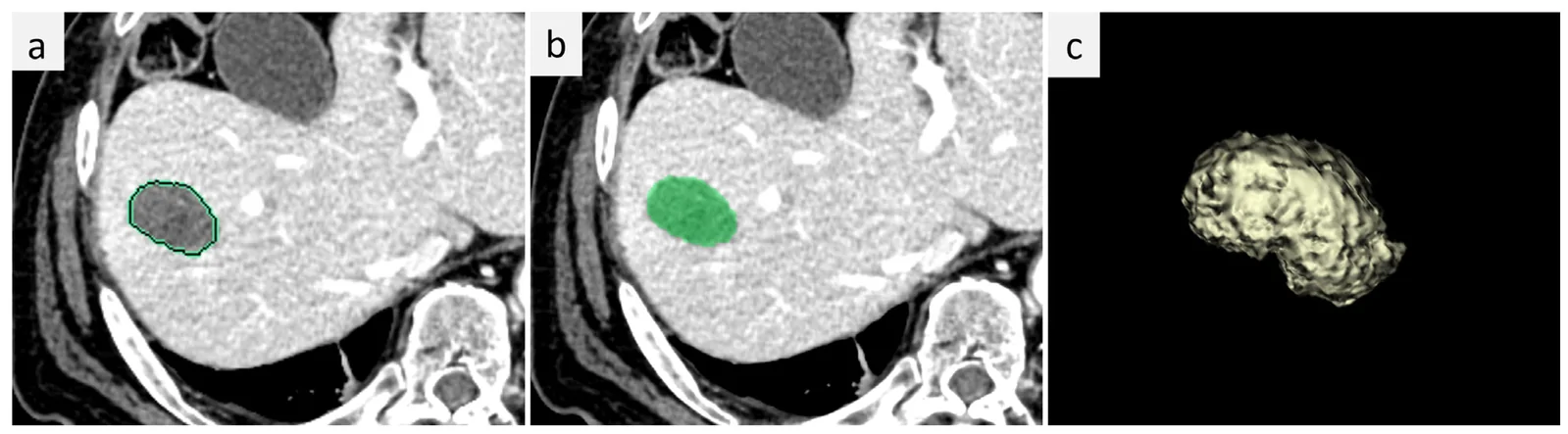
3D volumetric CT measurements of the ablated area in the liver ((**a**) manually traced image, (**b**) automatically summed image, (**c**) 3D image). The green contour in (**a**) indicates the manually traced boundary, and the green shaded area in (**b**) represents the corresponding segmented volume.

3D volumetric CT measurements of the ablated area in the liver were performed on portal-phase images with a slice thickness of 1 mm using a volume analyzer software program (SYNAPSE VINCENT, version 7; Fujifilm Corporation, Tokyo, Japan) on a workstation. The volume of the ablated area (AAV) on the follow-up CT images obtained within 1 week and around 6 months after RFA was initially measured by a third-year medical student (Y.F.) who was blinded to any clinical information of the patients. All measurements were subsequently reviewed by one radiologist (M.H., 10 years of experience in body imaging), and minor corrections were made when necessary. The final measurements were established by consensus. The contours of the ablated area in the liver were manually traced at regular intervals (every few slices), and the traced ablated area was automatically summed to calculate AAV (Figure 1). Based on the AAV within 1 week and around 6 months after RFA, the reduction rate of AAV was calculated using the following formula: (AAV within 1 week after RFA–AAV around 6 months after RFA)/AAV within 1 week after RFA × 100. The reduction rate of AAV was correlated with the clinical data and compared among the categories of Child-Pugh classification, mALBI grade, FIB-4 index, and lesion location. One radiologist (M.H.) also assessed the location of the lesions and measured the maximum diameter of the lesions on pre-RFA CT images or on T1-weighted gradient-echo gadoxetic acid-enhanced hepatobiliary phase images of MRI when CT was not available.

### 2.5. Statistical Analyses

All statistical analyses were performed using SPSS (ver. 27.0, IBM). Normality was tested using the Shapiro–Wilk test. Because some patients contributed multiple lesions, all lesion-level analyses were performed using generalized estimating equations (GEE) with patient identification as the clustering variable. Group comparisons according to Child–Pugh classification, mALBI grade, FIB-4 index, and tumor location were performed using GEE. Pairwise comparisons were adjusted using the Bonferroni method when appropriate. Univariable GEE analyses were conducted to evaluate the associations between the AAV reduction rate and clinical parameters. Variables with *p* values < 0.05 in the univariable analyses were considered for inclusion in the multivariable GEE analysis. Variance inflation factors (VIFs) were calculated to assess multicollinearity among the variables included in the final multivariable GEE model. *p* values of <0.05 were considered to indicate statistical significance.

## 3. Results

A total of 41 patients with 53 lesions met our inclusion criteria and were enrolled in this study (males, *n* = 24; females, *n* = 17; median age, 76 [range, 38–88] years). The patient-level and lesion-level characteristics are summarized in Table 1. The median size of the lesions that underwent RFA was 14 mm (range, 7–26 mm). The lesion locations were as follows: lateral segment (*n* = 11), medial segment (*n* = 2), anterior segment (*n* = 22), and posterior segment (*n* = 18). Because only two lesions were located in the medial segment, the medial and anterior segments were combined for subsequent statistical analyses. The median AAV within 1 week and around 6 months after RFA was 17.5 mL (range, 5.0–83.2 mL) and 5.4 mL (range, 1.1–30.2 mL), respectively. The median interval between the first and second follow-up CT examinations was 178 days (range, 136–220 days). The mean reduction rate of AAV was 67.3 ± 12.6% (range, 37.0–95.4%).

**Figure 2:**
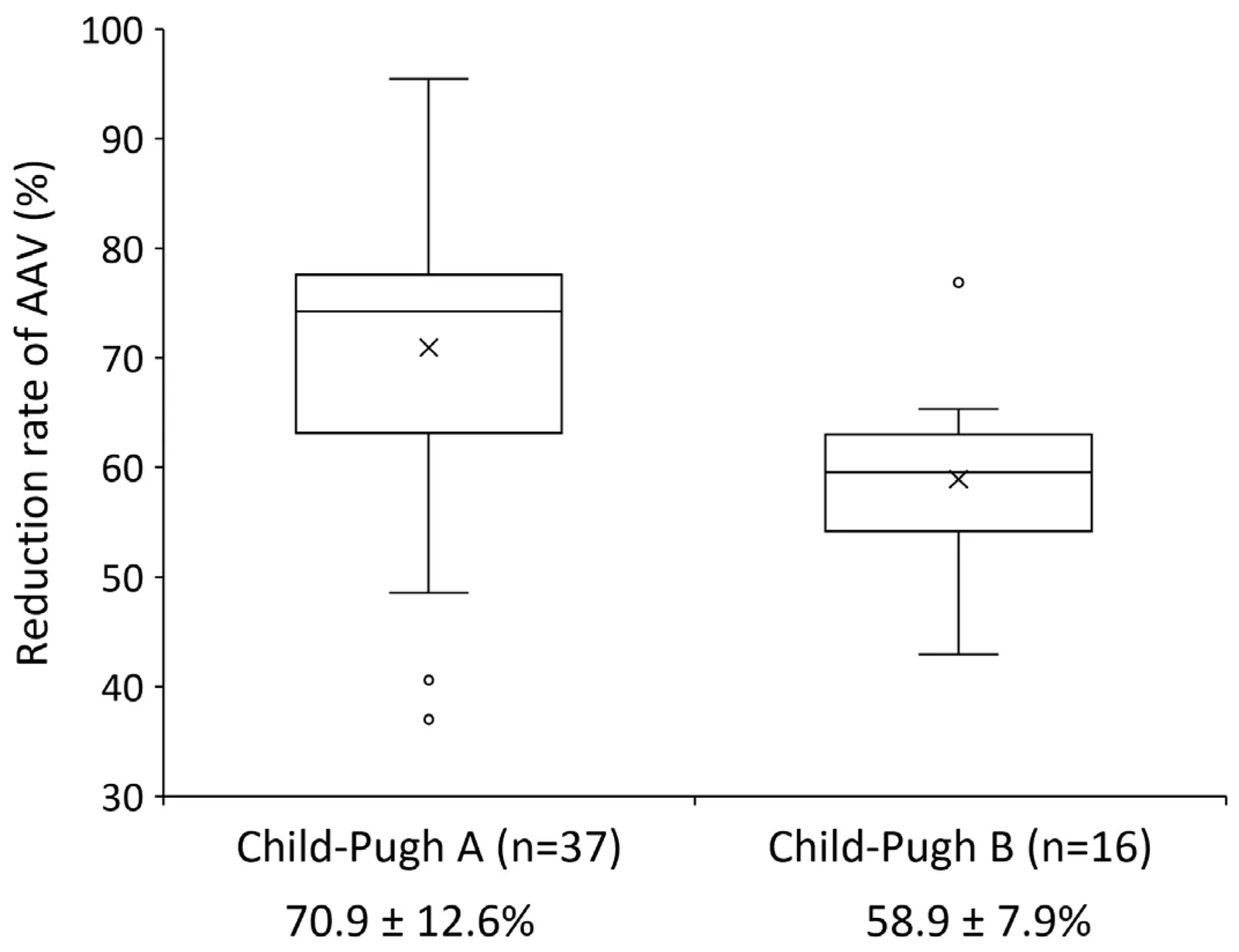
Boxplot of the reduction rate of AAV according to Child-Pugh classification. The reduction rate of AAV in Child-Pugh B group was significantly lower than in Child-Pugh A group (*p* < 0.001). The × symbol indicates the mean value, and open circles indicate outliers.

**Figure 3:**
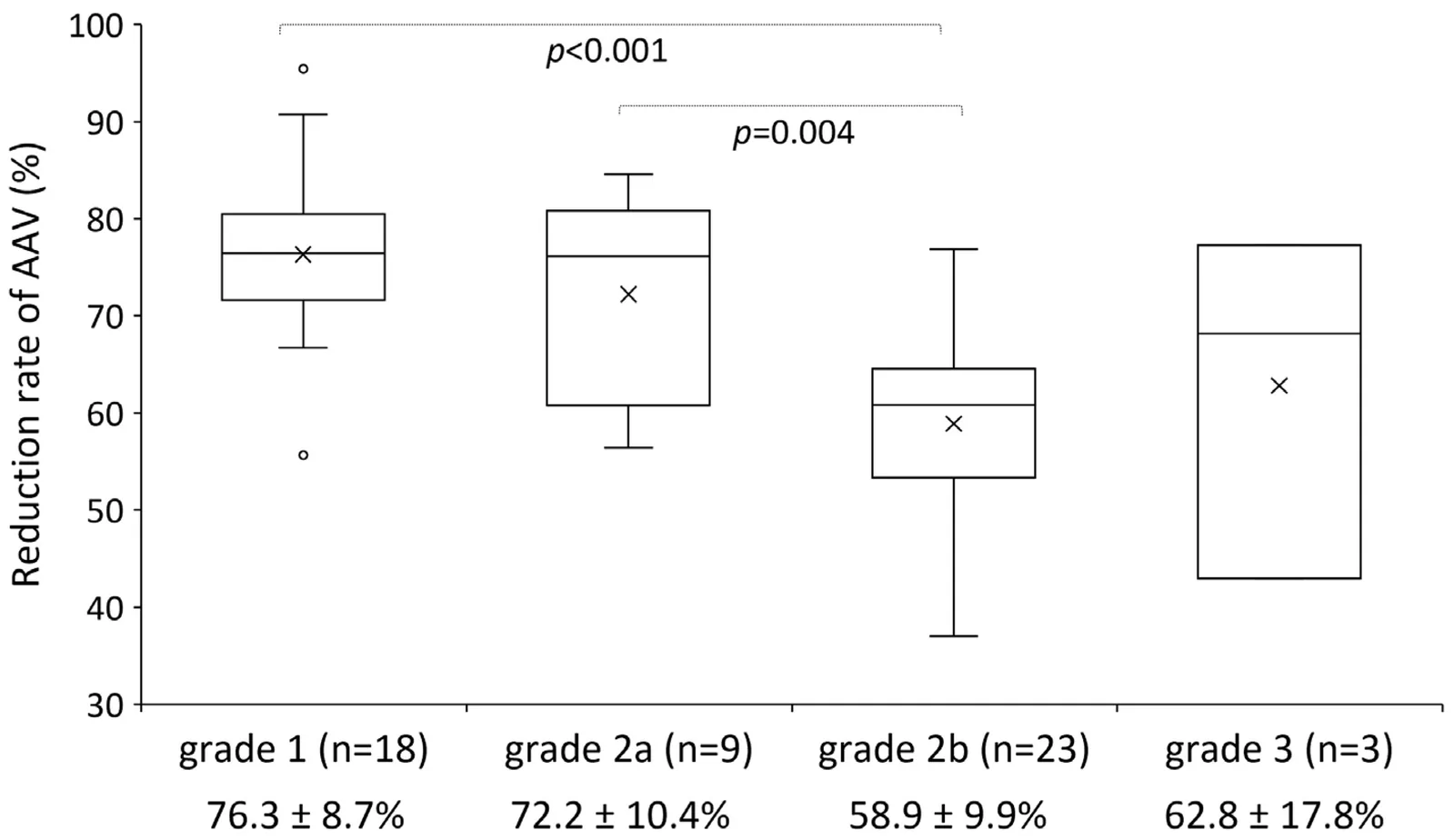
Boxplot of the reduction rate of AAV according to mALBI grade. The reduction rate of AAV in grade 2b group was significantly lower than in grade 1 and grade 2a groups (*p* < 0.001 and *p* = 0.004, respectively). The × symbol indicates the mean value, and open circles indicate outliers.

**Figure 4:**
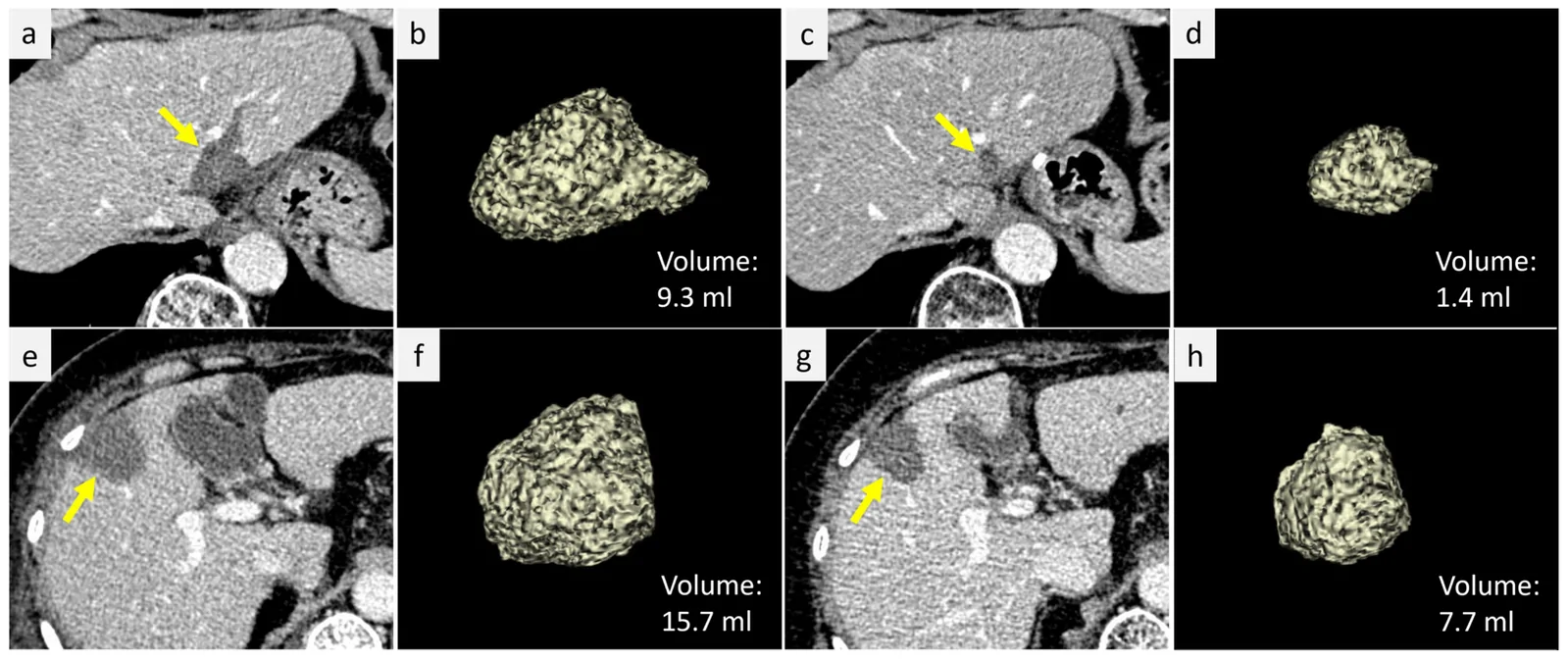
Representative images of the ablated area in the liver (yellow arrows): contrast-enhanced CT images obtained within 1 week (**a**,**e**) and around 6 months (**c**,**g**) after RFA and 3D images within 1 week (**b**,**f**) and around 6 months (**d**,**h**) after RFA. In a 72-year-old man with preserved liver function (Child-Pugh A and ALBI score of −2.59/mALBI grade 2a) (**a**–**d**), the reduction rate of AAV was 84.6%. In an 81-year-old woman with impaired liver function (Child-Pugh B and ALBI score of −1.46/mALBI grade 2b) (**e**–**h**), the reduction rate of AAV was 51.0%.

The comparison of the reduction rate of AAV according to the Child-Pugh classification and mALBI grade is shown in Figure 2 and Figure 3. The AAV reduction rate in the Child-Pugh B group was significantly lower than that in the Child-Pugh A group (58.9 ± 7.9% vs. 70.9 ± 12.6%, *p* < 0.001). In addition, the reduction rate of AAV in the mALBI grade 2b group (58.9 ± 9.9%) was significantly lower than in the grade 1 (76.3 ± 8.7%) and grade 2a (72.2 ± 10.4%) groups (*p* < 0.001 and *p* = 0.004, respectively). Representative images of the ablated liver area for different Child-Pugh classifications and mALBI grades are shown in Figure 4.

**Figure 5:**
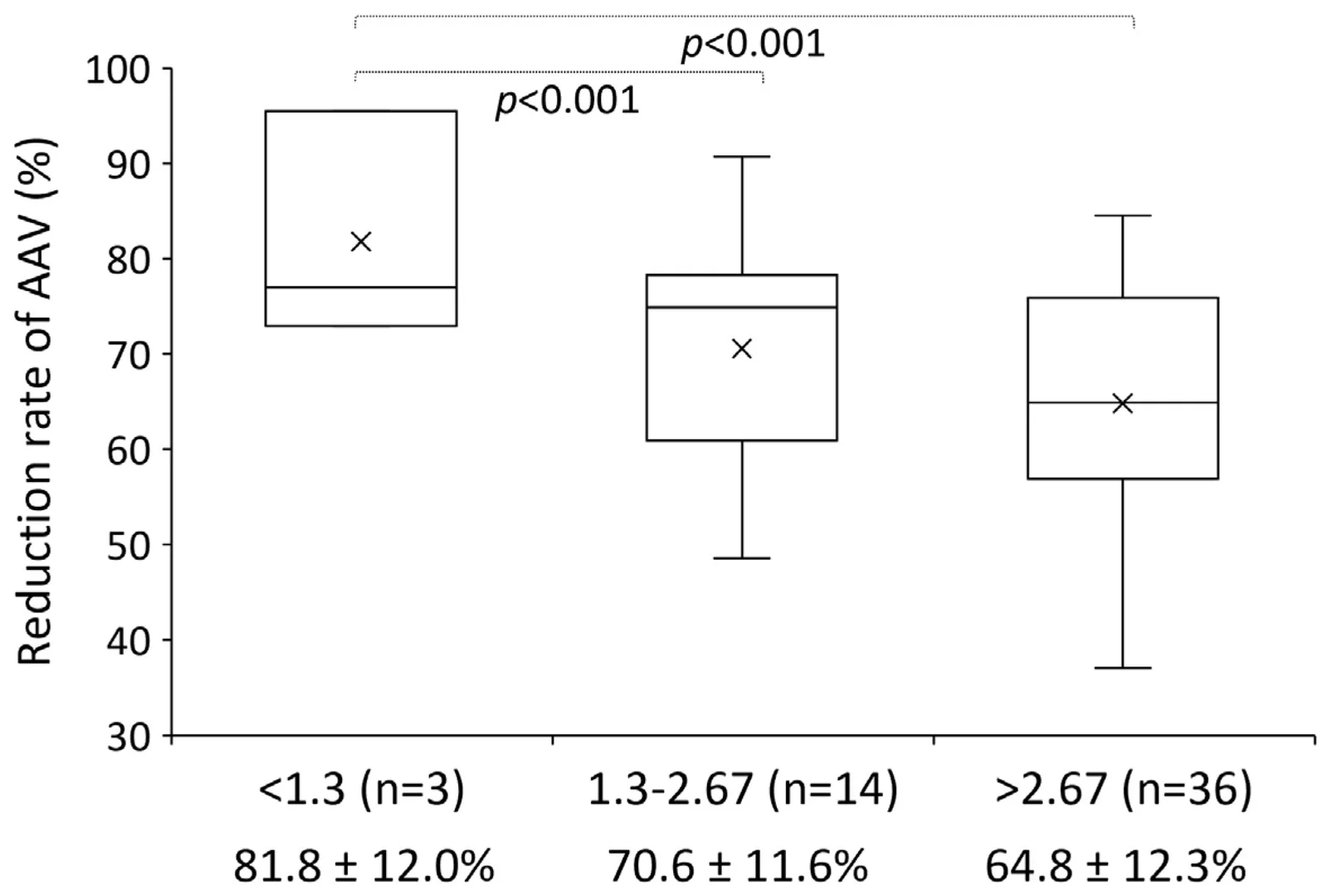
Boxplot of the reduction rate of AAV according to FIB-4 index. The reduction rate of AAV in FIB-4 < 1.3 group was significantly higher than in FIB-4 1.3 to ≤2.67 and FIB-4 > 2.67 groups (both *p* < 0.001). The × symbol indicates the mean value.

The comparison among the three FIB-4 index groups revealed significant differences in the reduction rate of AAV (FIB-4 < 1.3, 81.8 ± 12.0%; FIB-4 1.3 to ≤2.67, 70.6 ± 11.6%; and FIB-4 > 2.67, 64.8 ± 12.3%; *p* < 0.001) (Figure 5). The AAV reduction rate in the FIB-4 < 1.3 group was significantly higher than those in both the FIB-4 1.3 to ≤2.67 group and the FIB-4 > 2.67 group (both *p* < 0.001).

**Figure 6:**
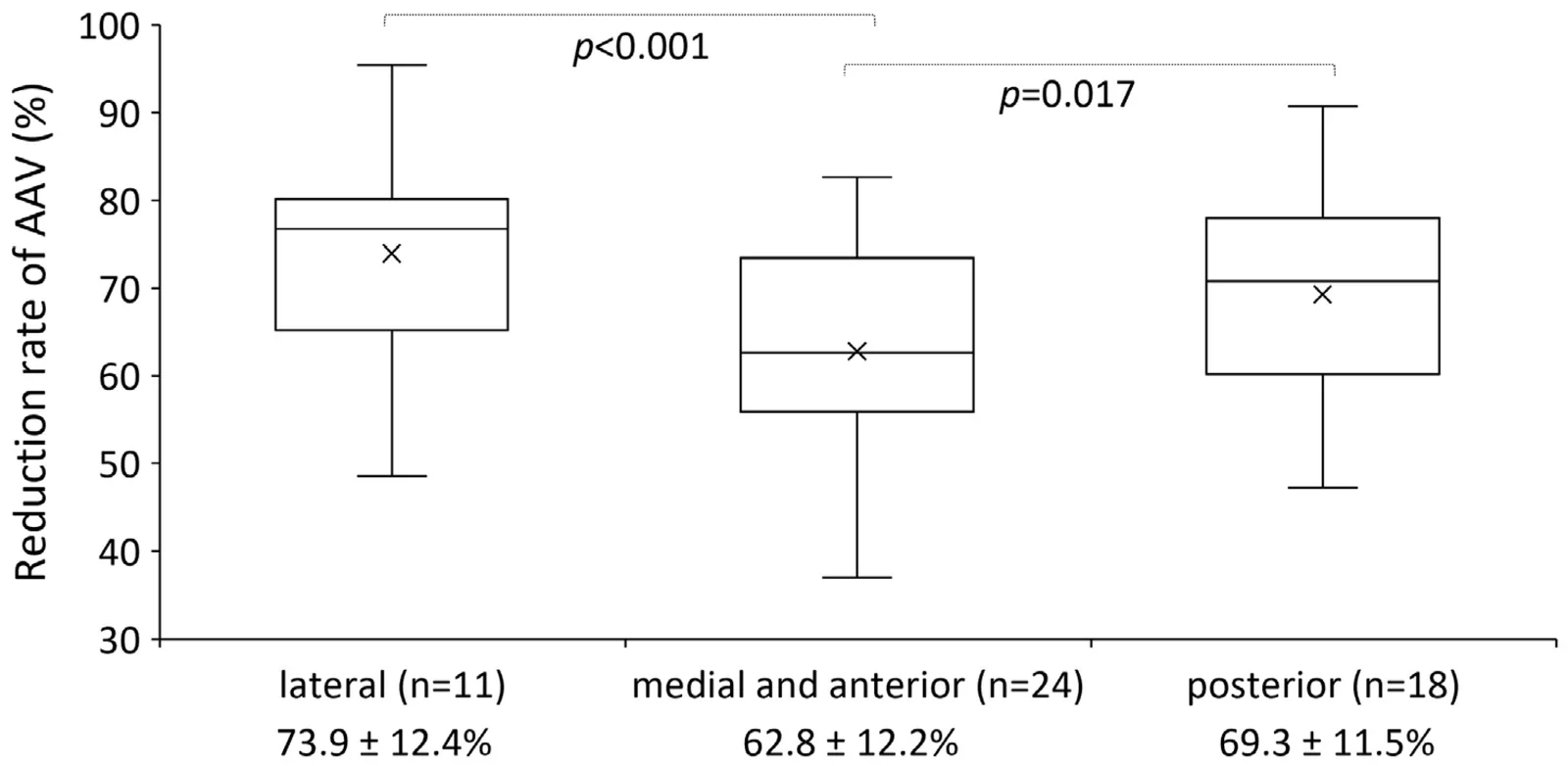
Boxplot of the reduction rate of AAV according to liver segments. The reduction rate of AAV in medial and anterior segments was significantly lower than in lateral and posterior segments (*p* < 0.001 and *p* = 0.017, respectively). The × symbol indicates the mean value.

A comparison of the AAV reduction rate according to lesion location is shown in Figure 6. The mean AAV reduction rate in each segment was as follows: lateral segment, 73.9 ± 12.4%; medial and anterior segments, 62.8 ± 12.2%; and posterior segment, 69.3 ± 11.5%. The comparison among the three segment groups revealed significant differences in the reduction rate of AAV (*p* < 0.001). The AAV reduction rate in the medial and anterior segments was significantly lower than that in the lateral segment (*p* < 0.001) and that in the posterior segment (*p* = 0.017).

The results of the univariable and multivariable GEE analyses are presented in Table 2. Univariable GEE analysis showed that the AAV reduction rate was significantly associated with ChE, albumin, T-Bil, prothrombin time, platelet count, and FIB-4 index (*p* < 0.05). Multivariable GEE analysis demonstrated that both albumin and T-Bil remained independently associated with the AAV reduction rate (both *p* < 0.001). The VIFs for albumin and T-Bil were both 1.044, indicating no evidence of problematic multicollinearity. Neither the interval between the first and second follow-up CT examinations (*β* = −0.043, 95% CI: −0.199 to 0.113, *p* = 0.587) nor the initial AAV measured within 1 week after RFA (*β* = −0.011, 95% CI: −0.367 to 0.345, *p* = 0.952) was significantly associated with the AAV reduction rate in the univariable GEE analysis.

## 4. Discussion

Our study findings showed that the reduction rate of AAV tended to be lower in patients with an impaired hepatic functional reserve. In addition, the liver fibrosis index and location of the ablated area in the liver were also found to be related to the reduction rate of AAV.

In RFA, the ablated area has been reported to shrink over time [9,15,16,17], probably because of regenerative recovery of the surrounding liver parenchyma. An experimental study has demonstrated the increased expression of proliferation markers in hepatocytes around the ablation zone [18], suggesting that shrinkage of the ablated area may be associated with liver regeneration.

In this study, patients with a Child-Pugh B or higher mALBI grade showed a significantly lower reduction rate of AAV. These findings suggest that the ablated area is less likely to shrink in patients with an impaired liver function, which probably reflects the diminished regenerative capacity of the liver. This is consistent with previous studies indicating that the liver function markedly affects liver regeneration [19,20,21].

Furthermore, the multivariable GEE analysis identified serum albumin and T-Bil levels as the factors independently associated with the reduction rate of AAV. Notably, both parameters are components of the ALBI score. Considering that the ALBI score is an objective and sensitive indicator of the liver function and has been extensively validated as a prognostic marker in HCC treatments, including RFA [22,23], our findings indicate that the liver function, as evaluated by mALBI grade, strongly influences the degree of shrinkage of the ablated area. Therefore, these results suggest that RFA in patients with severe liver dysfunction carries the risk of insufficient liver regeneration after ablation.

In this study, the AAV reduction rate showed a significant correlation with the FIB-4 index. In addition, a significant difference in the FIB-4 index was observed among the three FIB-4 range groups, and pairwise comparisons demonstrated significantly higher AAV reduction rates in the FIB-4 < 1.3 group than in the FIB-4 1.3 to ≤2.67 group and FIB-4 > 2.67 groups. These findings suggest that the ablated area may be less reducible even in patients with advanced liver fibrosis. Our findings are consistent with those of previous studies that demonstrated that the regenerative capacity of the liver is often diminished in patients with liver cirrhosis [24,25].

Among the liver segments, the medial and anterior segments exhibited the lowest AAV reduction rate, whereas the lateral segment showed a relatively high reduction rate. The medial and anterior segments are included in the middle hepatic venous (MHV) drainage area and have been reported to exhibit significant atrophy in cirrhotic livers [26]. The MHV, with the smallest diameter [26], is more susceptible to compression by regenerative nodules and fibrosis in cirrhosis, leading to impaired hepatic venous outflow and subsequent reduction in portal venous inflow [27]. Therefore, the lower reduction rate of AAV in this area may be explained by diminished hepatic regenerative capacity resulting from hepatocyte loss and progression of hepatic fibrosis due to decreased portal venous flow [28]. In contrast, it is well recognized that the lateral segment tends to undergo compensatory hypertrophy in chronic liver disease and cirrhosis [26,29,30], which may reflect relatively preserved portal venous flow. Moreover, a recent study demonstrated an increase in the lateral segment volume following RFA of the liver, suggesting a regenerative response [16]. Thus, the greater shrinkage of the ablated area in this segment may be attributed to the preserved hepatic regenerative capacity.

Long-term follow-up CT examinations after RFA are necessary to evaluate treatment efficacy and potential complications, detect early signs of local tumor recurrence, and monitor changes in the ablation zone [31]. Previous studies have shown that the appearance of the ablation zone on CT varies considerably depending on the time elapsed after RFA, the specific local environment, and the presence of accompanying complications [32,33]. However, regarding changes in the size of the ablation zone, interpatient differences in the degree of ablation zone shrinkage and the factors influencing them remain unclear. The present study identified several factors that significantly affected ablation zone shrinkage, including liver function, liver fibrosis index, and the location of the ablated area. Understanding these factors may help guide ablation strategies and optimize post-ablation surveillance, ultimately contributing to improved RFA patient management. In addition, consideration of these factors may facilitate more accurate interpretation of follow-up CT findings, because progressive shrinkage of the ablation zone may lead to underestimation of the original ablative margin on delayed CT examinations. Although our findings have important implications for post-RFA imaging assessment, they should be interpreted in the context of RFA. Because microwave ablation (MWA) has different ablation characteristics, including faster heating and reduced susceptibility to the heat-sink effect [7], the present findings may not be directly applicable to MWA. Further comparative studies are warranted to clarify whether ablation zone shrinkage differs between RFA and MWA.

The present study has several limitations. First, as this was a retrospective study with a relatively small number of patients, selection bias may have been inevitable. Second, volumetric measurements were obtained through a consensus workflow rather than independent assessment by two observers. Therefore, formal interobserver reproducibility was not assessed. In addition, volumetric measurements were performed using manual tracing. Future studies incorporating independent measurements by multiple observers and deformable image registration are warranted to improve the reproducibility and accuracy of longitudinal volumetric assessment. Third, the potential effects of prior local treatments for HCC, including hepatectomy, transcatheter arterial therapies, previous RFA, and radiotherapy, were not incorporated into the statistical analysis. These treatments may alter regional portal perfusion and induce localized fibrosis, potentially influencing the reduction rate of AAV. However, because treatment histories were highly heterogeneous with respect to treatment type, number, timing, and anatomical location, and because of the limited sample size, their effects could not be reliably evaluated in the present study. Additionally, other factors that could influence liver regeneration and ablation zone shrinkage, including body mass index, hepatic steatosis, portal pressure, and inflammatory responses after RFA [18,34,35], were not evaluated in the present study. Further studies incorporating inflammatory biomarkers are warranted to clarify their potential contribution to ablation zone shrinkage. Finally, the follow-up period was limited to approximately 6 months after RFA, as follow-up CT examinations at our institution were often restricted to this duration. Although follow-up intervals varied among cases, no significant association was observed between the follow-up interval and the AAV reduction rate. Further studies with a larger number of patients and extended follow-up are warranted to validate our results and clarify the long-term clinical implications of changes in the ablation zone.

## 5. Conclusions

The reduction rate of AAV tended to be lower in patients with impaired liver function and advanced liver fibrosis. The limited shrinkage of the ablated liver area over time may reflect a compromised hepatic regenerative capacity.

## Figures and Tables

**Table 1:** Patient and lesion characteristics.

Variable	Value
Patient characteristics (*n* = 41)	
Age (y)	76 (71–81)
Male/female (*n*)	24/17
Lesions per patient 1/2/3 (*n*)	31/8/2
Prior hepatectomy, *n* (%)	19 (46.3)
Prior TACE, *n* (%)	21 (51.2)
Prior TAE, *n* (%)	3 (7.3)
Prior TAI, *n* (%)	8 (19.5)
Prior RFA, *n* (%)	16 (39.0)
Prior radiotherapy, *n* (%)	5 (12.2)
Lesion characteristics (*n* = 53 lesions)	
Lesion size (mm)	14 (7–26)
Lesion location, lateral/medial/anterior/posterior (*n*)	11/2/22/18
AST (IU/L)	34 (26–48)
ALT (IU/L)	23 (16–38)
LDH (IU/L)	218 (203–242)
ALP (IU/L)	209 (104–355)
ChE (IU/L)	194 ± 67
Albumin (g/L)	35.6 ± 6.3
T-Bil (µmol/L)	12.0 (8.6–17.1)
D-Bil (µmol/L)	5.1 (3.4–6.8)
Prothrombin time (%)	88.1 (77.3–99.1)
Platelet count (×10^9^/L)	141 ± 62
FIB-4 index	3.83 (2.54–5.79)
Child-Pugh class A/B (*n*)	37/16
mALBI grade 1/2a/2b/3 (*n*)	18/9/23/3

Data are mean ± standard deviation or median with 25th and 75th percentiles in parentheses. TACE, transcatheter arterial chemoembolization; TAE, transcatheter arterial embolization; TAI, transcatheter arterial infusion chemotherapy; RFA, radiofrequency ablation; AST, aspartate aminotransferase; ALT, alanine aminotransferase; LDH, lactate dehydrogenase; ALP, alkaline phosphatase; ChE, cholinesterase; T-Bil, total bilirubin; D-Bil, direct bilirubin; FIB-4, fibrosis-4; mALBI, modified albumin-bilirubin. Laboratory data were recorded at the time of each RFA session (53 lesions) and may include repeated measurements from the same patient. Patients may have undergone more than one type of prior treatment.

**Table 2:** Univariable and multivariable GEE analyses of the association between reduction rate of AAV and clinical parameters.

	Univariable GEE			Multivariable GEE		
	*β*	95% CI	*p*	*β*	95% CI	*p*
Age (y)	0.018	−0.393 to 0.429	0.931			
AST (IU/L)	−0.135	−0.280 to 0.010	0.068			
ALT (IU/L)	−0.079	−0.232 to 0.073	0.308			
LDH (IU/L)	−0.040	−0.120 to 0.040	0.329			
ALP (IU/L)	−0.015	−0.035 to 0.005	0.141			
ChE (IU/L)	0.082	0.029 to 0.135	0.002			
Albumin (g/L)	11.579	7.083 to 16.075	<0.001	10.424	6.098 to 14.750	<0.001
T-Bil (µmol/L)	−12.119	−18.625 to −5.613	<0.001	−9.575	−15.101 to −4.050	<0.001
Prothrombin time (%)	0.303	0.138 to 0.469	<0.001			
Platelet count (×10^9^/L)	0.607	0.026 to 1.188	0.041			
FIB-4 index	−0.901	−1.497 to −0.305	0.003			

GEE, generalized estimating equations; AAV, ablated area volume; AST, aspartate aminotransferase; ALT, alanine aminotransferase; LDH, lactate dehydrogenase; ALP, alkaline phosphatase; ChE, cholinesterase; T-Bil, total bilirubin; FIB-4, fibrosis-4.

## Data Availability

The datasets generated or analyzed during the study are available from the corresponding author upon reasonable request due to ethical restrictions; access requires approval from the institutional ethics committee.

## Abbreviations

The following abbreviations are used in this manuscript:

HCC	Hepatocellular carcinoma
RFA	Radiofrequency ablation
TACE	Transcatheter arterial chemoembolization
TAE	Transcatheter arterial embolization
TAI	Transcatheter arterial infusion chemotherapy
MWA	Microwave ablation
AAV	Ablated area volume
AST	Aspartate aminotransferase
ALT	Alanine aminotransferase
LDH	Lactate dehydrogenase
ALP	Alkaline phosphatase
ChE	Cholinesterase
T-Bil	Total bilirubin
D-Bil	Direct bilirubin
FIB-4	Fibrosis-4
mALBI	Modified albumin-bilirubin
GEE	Generalized estimating equations
VIFs	Variance inflation factors
